# Performance and emissions of a diesel engine fueled by coal-based diesel fuels and their blends with polyoxymethylene dimethyl ethers

**DOI:** 10.1038/s41598-023-28283-y

**Published:** 2023-01-19

**Authors:** Yuwei Zhao, Ting Li, Tianlin Niu, Wenxiu Zheng, Yijing Xie, Weibo E

**Affiliations:** Air and Missile Defense College, Air Force Engineering University, Xi’an, 710051 People’s Republic of China

**Keywords:** Energy science and technology, Engineering

## Abstract

The objective of this study was to investigate the performance and emissions of a diesel engine fueled by coal-based diesel fuels and their blends with oxygenated fuel polyoxymethylene dimethyl ethers (PODEn). First, coal-based Fischer–Tropsch (FT) diesel fuel was blended with hydrogenated diesel fuel at three volume ratios of 40%/60%, 50%/50%, and 60%/40%, denoted as T6W4, T5W5, and T4W6, respectively. Then, PODEn were added into the T4W6 fuel with the volume ratios of 10%, 20%, and 30% to evaluate its effects on the performance and emissions of a coal-based diesel engine. The results showed that the output torques and powers of the three coal-based diesel blends were slightly lower than those of the petroleum diesel fuel. The brake specific fuel consumption (BSFC) of the coal-based diesel fuels was almost the same as that of the petroleum diesel fuel. The brake thermal efficiencies (BTE) of the coal-based diesel blends were slightly lower than that of the petroleum diesel fuel, and the maximum reduction was 1.59%. The pollutant emissions of T5W5 were the closest to those of petroleum diesel fuel. The nitrogen oxides (NOx) emissions of T4W6 were lower, with a maximum decrease of 11.18% compared with the petroleum diesel. The carbon monoxide (CO) and hydrocarbon (HC) emissions of T6W4 were the highest, with maximum increases of 36.79% and 29.05%, respectively. The smoke emissions of T4W6 and T6W4 were higher than those of petroleum diesel fuel. Adding PODEn into T4W6 lowered the engine power and torque but increased the BSFC and BTE. The output torque and power of the diesel engine were further reduced when PODEn were blended with T4W6, with the maximum reductions of 17.76% and 16.96%, respectively. With an increase in the PODEn blending ratio, BSFC and BTE increased gradually, and the maximum increase in the BTE was 1.57%. Blending PODEn with the fuel effectively improved the emission characteristics of the coal-based diesel fuels. The NOx emissions increased slightly, but the emissions of HC, CO, and smoke were reduced significantly, with maximum reductions of 24.42%, 31.67%, and 82.35%, respectively.

## Introduction

Currently, the large number of diesel engines consume a massive amount of fossil energy, resulting in energy shortages and environmental pollution. In China, coal is the primary form of energy consumption due to the national energy structure. Therefore, burning diesel and oxygenated fuels produced from coal in diesel engines can not only promote the clean utilization of coal resources but also reduce the dependence of overseas petroleum to a certain extent. Generally, coal-based diesel fuels can be produced by direct and indirect liquefication of coal^1–3^. In addition, coal can be used to produce oxygenated fuels with high cetane numbers, such as dimethyl ether (DME)^4,5^ and polyoxymethylene dimethyl ethers (PODEn)^6–8^, which are suitable for diesel engines.

FT diesel produced by indirect coal liquefication has attracted widespread attention in recent years due to its various advantages, including a high cetane number (≥ 70), good atomization characteristics, and low aromatic hydrocarbon content^9,10^. Gill et al.^11^ summarized the combustion and emission characteristics of diesel engines fueled with FT diesel. The results showed that burning FT diesel fuel can simultaneously reduce both the particulate matter (PM) and NOx emissions and improve the thermal efficiency and combustion noise. Hydrogenated diesel produced by coal hydrogenation conversion technology has better lubricity than petroleum diesel, and the physical and chemical properties, such as the kinematic viscosity and condensation point, are similar to those of 0^#^ petroleum diesel^12^. Zuo et al.^13^ investigated the combustion and emission characteristics of partially hydrogenated biodiesel (PHB)–ethanol–diesel ternary blends and PHB–diesel binary blends on a turbo-charged, common-rail diesel engine. The results showed that no significant variations of the equivalent specific fuel consumption occurred between the binary and ternary blends. Compared to diesel, the binary blends lowered the peak cylinder pressure and maximum heat release rate in the main combustion stage, whereas ternary blends exhibited the opposite phenomenon. Ternary blends generated higher HC and CO emissions but lower smoke emissions than binary blends. Higher sulfur dioxide (SO_2_), formaldehyde (HCHO), and carbon dioxide (CO_2_) emissions but lower ethylene (C_2_H_4_) and ammonia (NH_3_) emissions were obtained for ternary blends than for binary blends.

Adding oxygenated fuels into diesel can improve the combustion process and thus reduce the PM emissions^14^. Jiao et al.^15^ studied the combustion characteristics, performance, and emissions of a turbocharged diesel engine fueled with diesel and methanol–FT diesel–biodiesel–diesel blends at various simulated altitudes. The results indicated that the use of the blends improved the engine power and economic efficiency at low-speed ranges at an altitude of 3500 m, but a dramatic reduction was observed at 5500 m. Compared with petroleum diesel, the PM emissions of the blends were markedly lower, while the NOx emissions were slightly higher at various altitudes. Moujdin et al.^16^ studied the performance and emission characteristics of a diesel engine fueled with hydrogenated diesel/hydrogen peroxide (H_2_O_2_) blends. The results showed that the emissions of CO, SO_2_, HC, and NOx were reduced as the H_2_O_2_ blending ratio slightly increased. Based on the experimental study, H_2_O_2_ blending ratios of 5% and 10% were recommended.

The discussion above indicates that both FT diesel and hydrogenated diesel can be used in diesel engines. Improvement of the fuel properties is still needed for the fuel to be better burned in diesel engines. However, due to the poor lubricity of FT diesel, additional lubricating additives need to be added. Hydrogenated diesel oil has good lubricity but poor emission characteristics. Therefore, FT diesel and hydrogenated diesel were blended to realize the complementary advantages of the two fuels. The feasibility of the application of coal-based diesel blends in diesel engines was verified. In addition, the combustion and emission characteristics of coal-based diesel fuels can be improved by blending a certain proportion of oxygenated fuel. Studies have shown that PODEn, a coal-based oxygenated fuel with a high oxygen content and cetane number, can significantly reduce the PM emissions, and there is no need to modify the engine when blending with a small proportion^17–20^. Thus, on the basis of studying the performance of FT diesel and hydrogenated diesel blends, a small proportion of PODEn were blended to improve the emission characteristics. In this study, the performance of a diesel engine fueled with coal-based diesel fuels was investigated, and the emission characteristics were improved by blending oxygenated fuel. The results can provide support for the large-scale promotion and application of coal-based diesel fuels in diesel engines.

Though the application of FT diesel has been widely studied in previous work, it is still difficult to use FT diesel solely in diesel engines due to its poor lubricity. Considering the better lubricity of hydrogenated diesel, it is predicted that mixtures of coal-based FT diesel and hydrogenated diesel may benefit the fuel supply system. However, the performance and emissions of diesel engines fueled with FT diesel/hydrogenated diesel blends are still unexplored. Therefore, the first main objective of this study was to preliminarily explore the feasibility of using FT diesel/hydrogenated diesel blends in diesel engines. The power performance, economy, and emissions of a high-pressure common-rail diesel engine were analyzed, when fueling with FT diesel/hydrogenated diesel blends at volume ratios of 40%/60%, 50%/50%, and 60%/40% (denoted as T6W4, T5W5, and T4W6), respectively.

After evaluating the feasibility of using FT diesel/hydrogenated diesel blends, the further optimization of the blends by fuel modifications is also considered^21^. Previous studies showed that coal-based PODEn are promising oxygenated additives for diesel engines due to their high oxygen content and cetane number^17–20,22,23^. Therefore, the effects of blending with PODEn with ratios of 10, 20, and 30 vol% on the performance and emissions of T4W6-fueled engines were examined. The results can provide new insights for the application of coal-based diesel fuels in diesel engines.

## Materials and experiments

### Experimental apparatus

A turbo-charged, inter-cooled, high-pressure common-rail diesel engine (Model IVECO SOFIM 8140.43S) was used in this experimental study. The specifications of the engine are shown in Table 1.

**Table 1:** Specifications of the test engine.

Parameter	Specification
Engine	In-line 4-cylinder, 4-stroke, turbo-charged, inter-cooled, high-pressure, common-rail
Bore × stroke	94.4 mm × 100 mm
Displacement	2.798 L
Compression ratio	18.5
Maximum power/speed	92 kW/3600 rpm
Maximum torque/speed	290 Nm/1800 rpm
Fuel injection system	Boshi EDCM 6.3

**Figure 1:**
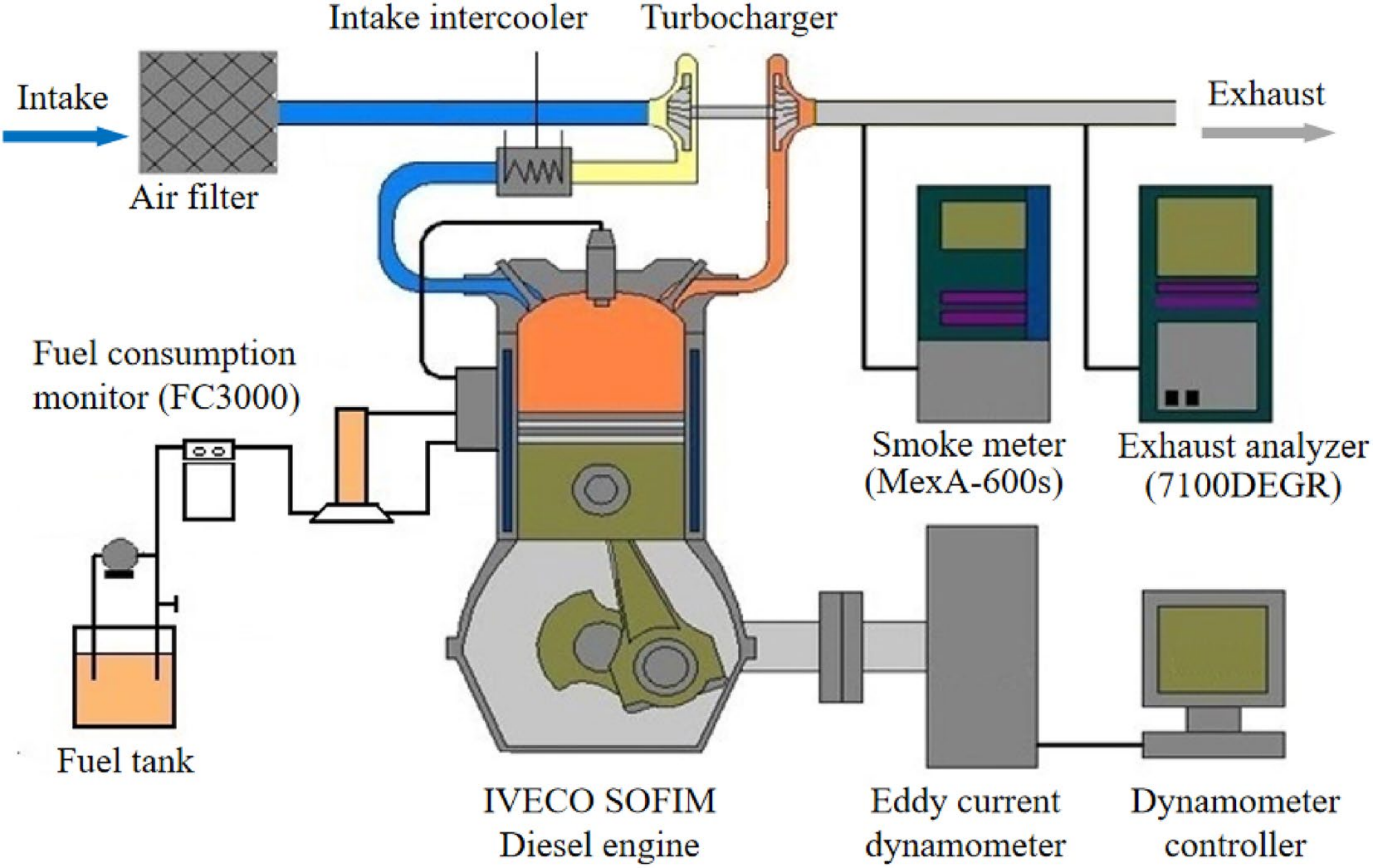
Schematic diagram of the testing apparatus.

The schematic diagram of the engine bench layout is shown in Fig. 1. The output torque and speed were controlled by an eddy current dynamometer (GW160, Xiangyi Power Co., Ltd.). The emissions of NOx, HC, and CO were measured by an exhaust gas analyzer (Horiba 7100DEGR). A smoke meter (Horiba MexA-600 s) was used to measure the exhaust smoke. The fuel consumption was measured by a mass flow meter (Siemens FC3000). The specifications of the testing apparatus were shown in Table 2. All the equipments and apparatus were calibrated before the tests in order to ensure the reliability of the experiment and the accuracy of the results. Each operating point was measured repeatedly to eliminate the uncertainty. The data were measured 5 times at the same operating condition after the engine was operating stably for more than 10 min, the uncertainty of individual parameters (Table 2) and the overall experimental uncertainty were calculated. The overall experimental uncertainty was 3.051%. All the experimental uncertainty was limited in 5%, which met the requirement of the engineering. The power and torque were tested at engine speeds of 1000, 1500, 2000, 2500, 3000, and 3600 r/min. Then, at a constant engine speed of 2500 r/min, seven brake mean effective pressures (BMEPs) from 0.18 to 1.26 MPa with a step of 0.18 MPa were adopted to study the economy and emission characteristics of the diesel engines fueled with various blends.

**Table 2:** Specifications of the testing apparatus.

Apparatus	Parameter	Specification		Uncertainty
		Range	Accuracy	
GW160	Torque	0–680 Nm	0.1 Nm	± 0.25%
	Speed	0–4300 rpm	1 rpm	± 0.15%
Horiba 7100DEGR	NOx	0–10,000 ppm	1 ppm	± 1.65%
	HC	0.00–5.00%	0.01%	± 0.28%
	CO	0.00–12.00%	0.01%	± 1.45%
	CO_2_	0–20%	0.02%	–
	O_2_	0–25%	0.01%	–
Horiba MexA-600 s	Opacity	0.00% ~ 99.9%	0.01%	± 1.13%
Siemens FC3000	fuel consumption	0–20 kg/h	0.01 kg/h	± 0.55%

### Test fuels

FT diesel was provided by Weilai Energy Chemical Co., Ltd., which is denoted as W in this paper. The hydrogenated diesel was provided by Tianyuan Chemical Co., Ltd., which is denoted as T. The high-purity PODEn were produced by Yuhuang Chemical Group. The diesel fuel used in the test was 0^#^ commercial petroleum diesel and is denoted as D. The physical and chemical properties of the test fuels are listed in Table 3. As observed, FT diesel and hydrogenated diesel complemented each other in terms of properties such as the cetane number, density, and lubricity. Since the mixtures of FT diesel and hydrogenated diesel at the volume ratio of 50%/50% had a close density to that of the petroleum diesel, mixtures with volume ratios of 40%/60%, 50%/50%, and 60%/40% were selected, which are denoted as T6W4, T5W5, and T4W6, respectively. In addition, three PODEn/T4W6 blended fuels were prepared with PODEn volume percentages of 10%, 20%, and 30%, which are denoted as T4W6-P10, T4W6-P20, and T4W6-P30, respectively. The main properties of the tested fuels are shown in Table 4. In this work, the fuel densities were measured by suspended densitometers and the lower heating values of FT diesel, hydrogenated diesel, PODEn and 0^#^ petroleum diesel were measured by an XRY-1C oxygen bomb calorimeter. The lower heating values of the blend fuels were calculated based on the lower heating values and densities of the test fuels.

**Table 3:** Physical and chemical properties of FT diesel, hydrogenated diesel, and 0^#^ petroleum diesel.

Property		Hydrogenated diesel (T)	FT diesel (W)	0^#^ petroleum diesel (D)
Flash point (°C)		41	64	67
Condensation Point (°C)		4	− 30	− 7
Density at 20 °C (kg/L)		0.872	0.769	0.819
Kinematic viscosity at 20 °C (mm^2^/s)		4.05	3.276	3.81
Lower heating value (MJ/kg)		42.116	43.992	41.864
Cetane number		46.6	78.4	51
Component	Saturated hydrocarbon (%)	78.3	91.8	–
	Aromatic hydrocarbon (%)	21.7	8.2	–

**Table 4:** Physical and chemical properties of the test fuels.

Denoted	Volume percentages	Density (kg/L)	Lower heating value (MJ/kg)	Cetane number	Kinematic viscosity at 20 °C (mm^2^/s)	Oxygen content (%)
D	100% petroleum diesel	0.819	41.86	51	3.81	0
T4W6	FT diesel:Hydrogenated diesel = 6:4	0.802	43.62	65.68	3.566	0
T5W5	FT diesel:Hydrogenated diesel = 5:5	0.815	43.28	62.5	3.642	0
T6W4	FT diesel:Hydrogenated diesel = 4:6	0.827	43.00	59.32	3.721	0
T4W6-P10	T4W6:PODE = 9:1	0.838	40.65	66.952	3.156	5.71
T4W6-P20	T4W6:PODE = 8:2	0.857	37.83	68.224	2.792	11.95
T4W6-P30	T4W6:PODE = 7:3	0.879	35.15	69.496	2.471	16.34

## Results and discussion

### Engine performance of coal-based diesel fuels

#### Power performance

**Figure 2:**
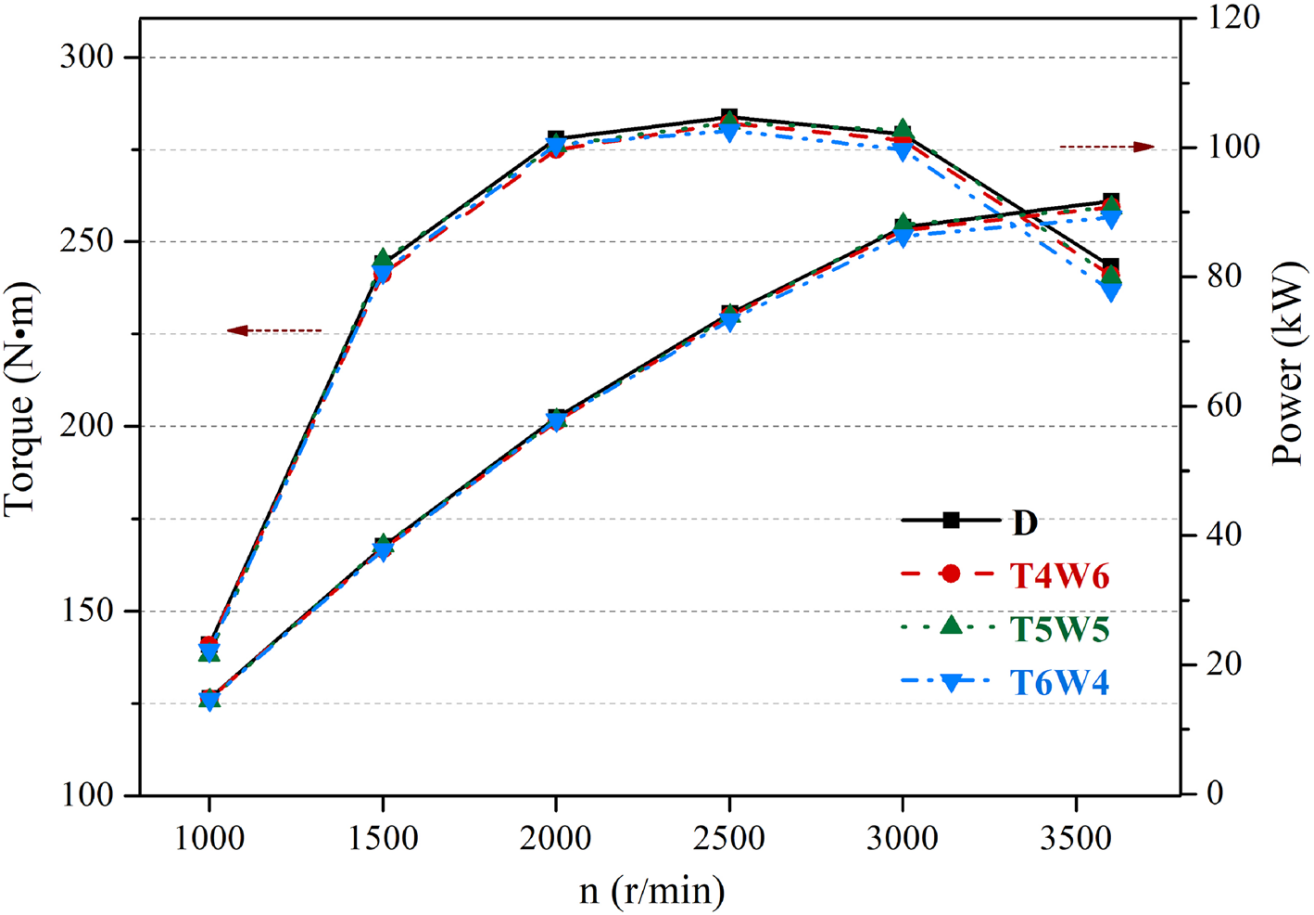
Power performance of three kinds of coal-based diesel blends and petroleum diesel.

The output torques and powers for the coal-based diesel blends with three ratios and petroleum diesel are illustrated in Fig. 2. The output powers of the three coal-based diesel blends with different proportions were almost the same as those of the petroleum diesel at low and medium speeds, while they were slightly lower than those of the petroleum diesel at high speeds. At the rated speed of 3600 r/min, in comparison to petroleum diesel fuel, the output powers of T4W6, T5W5, and T6W4 decreased by 0.98%, 1.09%, and 2.71%, respectively. This was because the physical and chemical properties of the three coal-based diesel blends were slightly different from those of petroleum diesel. The spray and atomization characteristics of the blends and the formation of combustible mixtures were affected^9,11^, resulting in lower combustion efficiencies. Meanwhile, the output power decreased with the increase in the hydrogenated diesel. The maximum torque of T4W6 was 1.29% lower than that of petroleum diesel. The torque of T5W5 was more similar to that of petroleum diesel than the other kinds of coal-based diesel blends because the physical and chemical properties of T5W5 were similar to those of petroleum diesel.

#### Fuel economy

**Figure 3:**
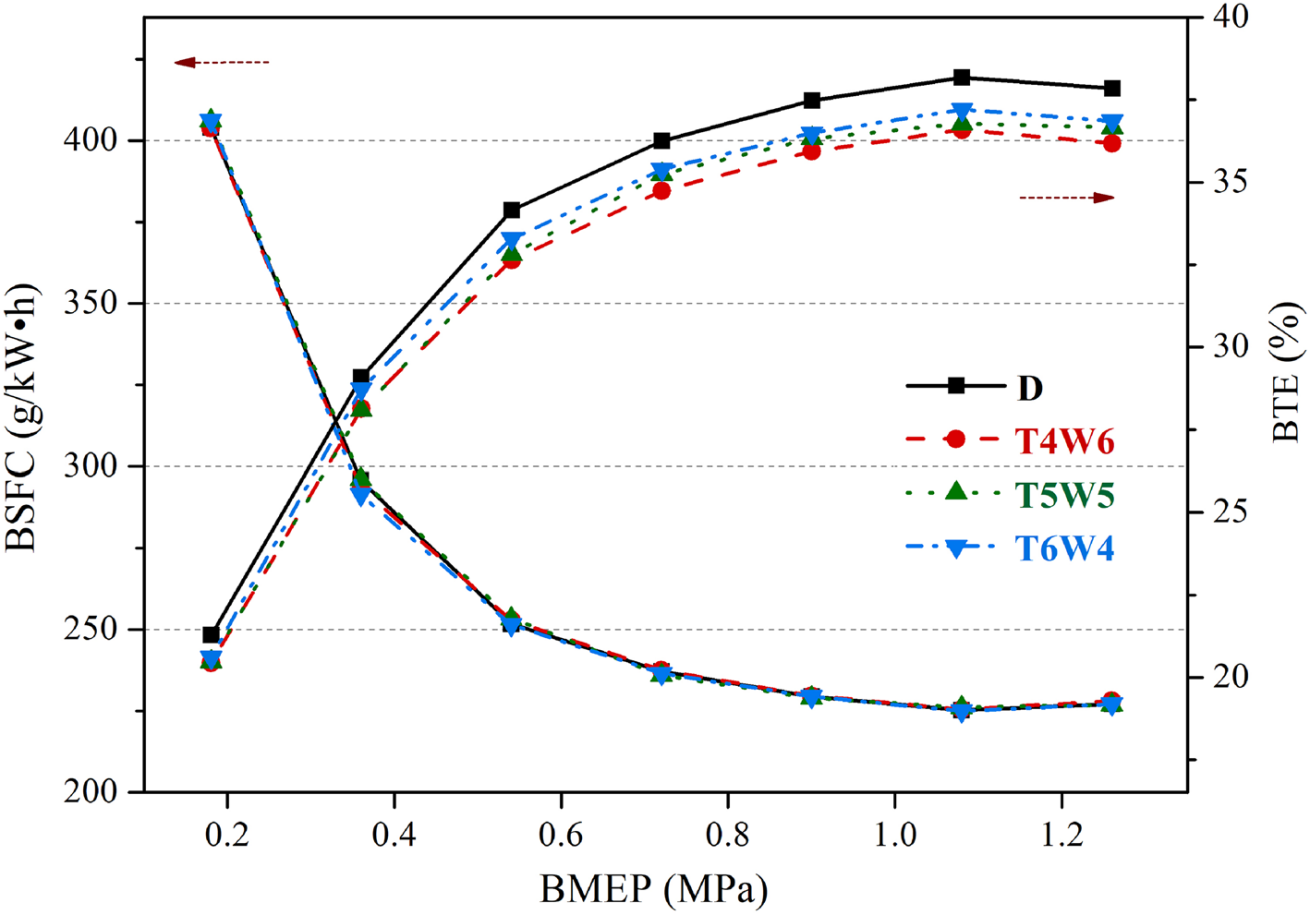
Fuel economies of three kinds of coal-based diesel blends and petroleum diesel.

The BSFC and BTE are two important indices to evaluate the fuel economy of an engine^24,25^. Figure 3 presents the BSFC and BTE values of the coal-based diesel blends and petroleum diesel at n = 2500 r/min. It can be seen that the BSFC values of the T4W6, T5W5, and T6W4 were almost the same as that of petroleum diesel, while the BTE values of the three coal-based diesel blends were lower. Compared with petroleum diesel, when BMEP = 1.08 MPa, the BTE values (absolute values) of T4W6, T5W5, and T6W4 decreased by 1.59%, 1.40%, and 0.97%, respectively.

The viscosities of the three kinds of coal-based diesel blends were different from that of the petroleum diesel^9,12,13^, which would affect the fuel spray characteristics. Thus, the formation of a combustible mixture in the cylinder would be affected. However, the evaporative properties of coal-based diesel fuels were better than those of petroleum diesel, which contributed to the formation of combustible mixtures. According to the test results, the former influence was dominant, making the BTE values of the three kinds of coal-based diesel blends lower than that of the petroleum diesel. Compared with hydrogenated diesel, the viscosity difference between FT diesel and petroleum diesel was larger. Therefore, T4W6 fuel had the lowest thermal efficiency due to its high FT diesel content. The spray characteristics of T4W6, T5W5, and T6W4 needed to be further verified by spray experiments.

#### Emission characteristics

**Figure 4:**
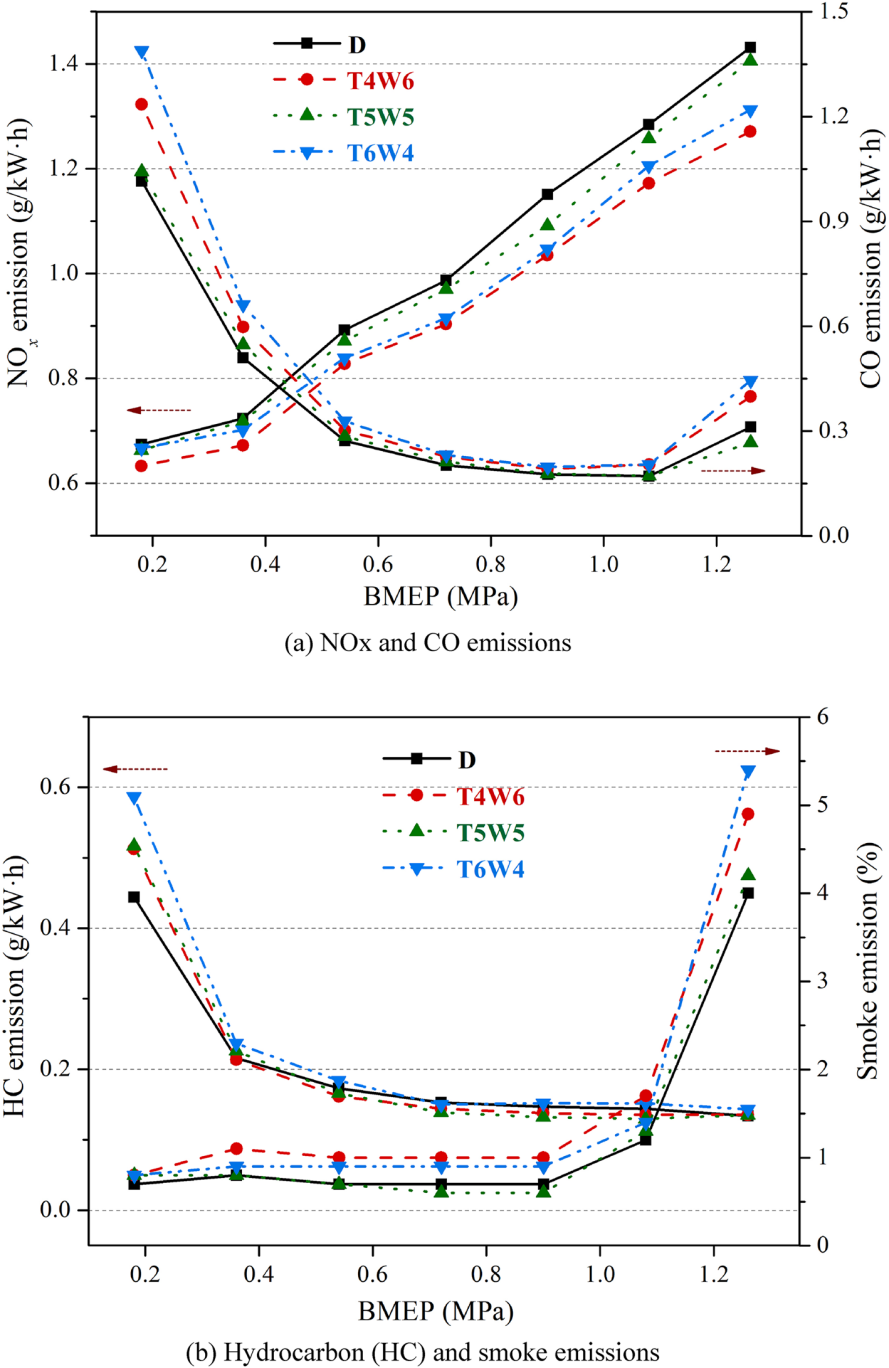
Emission characteristics of three kinds of coal-based diesel blends and petroleum diesel.

Figure 4 shows the emission characteristics of three kinds of coal-based diesel blends and petroleum diesel at n = 2500 r/min. As can be seen from Fig. 4a, the NOx emissions of T5W5 were close to that of petroleum diesel, while the NOx emissions of T4W6 and T6W4 were lower. When BMEP = 1.26 MPa, compared with petroleum diesel, the NOx emissions of T5W5 were reduced by 1.81%, while the NOx emissions of T4W6 and T6W4 were reduced by 11.18% and 8.29%, respectively. In diesel engines, nitric oxide (NO) is the predominant NOx produced in the combustion chamber. It is generally accepted that high NO formation results from high temperatures, high oxygen concentrations, and longer residence time under high temperature condition^26,27^. FT diesel had a high cetane number but a low density and latent heat of volume vaporization, while hydrogenated diesel had low cetane number but a high density and latent heat of volume vaporization. When they were blended at a volume ratio of 1:1, the cetane number and density of the T5W5 fuel were similar to those of the petroleum diesel, so the NOx emissions of T5W5 were similar to those of petroleum diesel. When they were mixed at a volume ratio of 6:4, the cetane number of the T4W6 fuel was higher than that of the petroleum diesel, and the ignition delay period could be shortened. Thus, the combustion temperature in the cylinder was decreased, which reduced the NOx emissions. When they were mixed at a volume ratio of 4:6, the cetane number of the T6W4 fuel was lower than that of the petroleum diesel, but the latent heat of volume vaporization was higher than that of the petroleum diesel. Therefore, the heat absorbed by evaporation after the fuel was injected into the cylinder increased, resulting in a relatively high temperature drop, which reduced the NOx emissions.

CO was mainly generated in low-temperature regions or fuel-rich regions^28^. As can be seen from Fig. 4a, with an increase in the load, the CO emissions first decreased and then increased. At low-load conditions, the in-cylinder temperature was relatively low, resulting in high CO emissions. With the increase in the load, the temperature in the cylinder increased and the CO emissions decreased. However, when the engine load further increased to the full-load conditions, the local fuel-rich regions in the combustion chamber increased, leading to a slight increase in the CO emissions. It can be observed that the CO emissions of T5W5 were close to those of petroleum diesel, but the CO emissions of T4W6 and T6W4 were higher than those of petroleum diesel. When BMEP = 0.18 MPa, the CO emissions of T4W6, T5W5, and T6W4 increased by 21.68%, 2.68%, and 36.79%, respectively. The spray characteristics of the three kinds of coal-based diesel blends were worse than those of the petroleum diesel fuel due to the physical and chemical property differences, resulting in an uneven gas mixture and high CO emissions. The physical and chemical properties of T5W5 were the most similar to those of petroleum diesel, which made the injection characteristics and mixture formation similar to those petroleum diesel, resulting in similar CO emissions.

HC emissions were mainly generated by the incomplete combustion in the thick or lean gas mixture in the cylinder^28,29^. Figure 4b shows that the HC emissions of the three kinds of coal-based diesel blends were higher than those of the petroleum diesel under low-load conditions, but the discrepancies of the HC emissions between the three coal-based diesel blends and the petroleum diesel became smaller under high-load conditions. When BMEP = 0.18 MPa, the HC emissions of T4W6, T5W5, and T6W4 were 15.44%, 16.27%, and 29.05% higher than those of petroleum diesel, respectively. The HC emissions of T6W4 were the highest. This was because of the high viscosity and density of the hydrogenated diesel, which made the spray quality of T6W4 poor and allowed a rich gas mixture to easily form, leading to an increase in the HC emissions.

Figure 4b also shows that the exhaust smoke of T5W5 was the most similar of the three coal-based diesel blends to that of the petroleum diesel. In some conditions, the exhaust smoke of T5W5 was even lower than that of petroleum diesel, while the smoke emissions of T4W6 and T6W4 were higher than those of petroleum diesel. When BMEP = 1.26 MPa, the exhaust smoke of T4W6, T5W5, and T6W4 was 22.5%, 5.0%, and 35.0% higher than that of petroleum diesel, respectively. The density, viscosity, and other physical and chemical properties of T5W5 were similar to those of petroleum diesel, causing similar spray and atomization characteristics. Thus, the exhaust smoke was similar to that of petroleum diesel. In addition, the exhaust smoke of T5W5 was lower than that of petroleum diesel in some conditions due to the low aromatic hydrocarbon content of T5W5. The fuel injection parameters of the original diesel engine were calibrated with neat petroleum diesel. The physical and chemical properties of T4W6 and T6W4 were slightly different from those of petroleum diesel. Thus, when the diesel engine was fueled with T4W6 and T6W4, the fuel spray and evaporation quality may have deteriorated, leading to an uneven air–fuel mixture and resulting in an increase in local fuel-rich regions and increased exhaust smoke emissions^30,31^.

### Effect of blending with PODEn on performance of coal-based diesel engine

By adopting the three different proportions of coal-based diesel fuels, it was found that the power performance of the three kinds of coal-based diesel blends were similar to that of the original diesel engine. However, the economies were slightly worse, and the emission characteristics had their own advantages. T5W5 achieved a similar performance to that of petroleum diesel, but the NOx emissions were relatively high. T4W6 could obtain the optimal NOx emissions, but the exhaust smoke was higher, and the thermal efficiency was lower. Research has shown that the addition of PODEn can effectively improve the exhaust smoke and thermal efficiency but also cause a slight increase in the NOx emissions^20^. In order to further improve the performance of coal-based diesel fuel, T4W6 was selected as the base oil in this study, and the effects of blending with PODEn on the engine power performance, economy, and emission characteristics were studied.

#### Power performance

**Figure 5:**
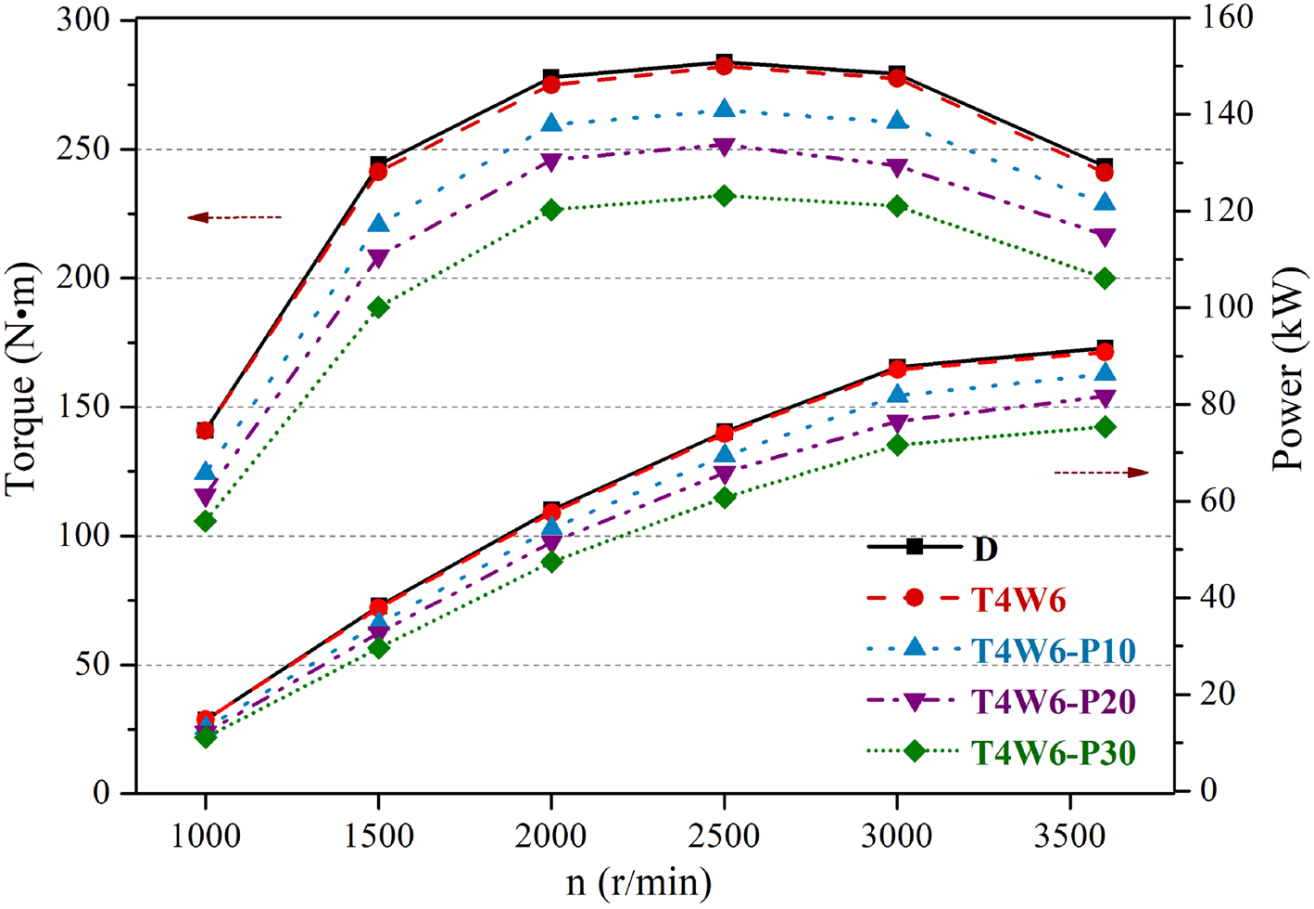
Effect of the polyoxymethylene dimethyl ethers (PODEn) blending ratio on the power performance of the diesel engine.

Figure 5 illustrates the output torques and powers of the diesel engine with different PODEn blending ratios. The torque and power decreased significantly at each speed when T4W6 was blended with PODEn, and the decrease extent increased with the increase in the PODEn blending ratio. At the maximum torque point (2500 r/min, full load), the output torques of T4W6-P10, T4W6-P20, and T4W6-P30 decreased by 6.06%, 10.74%, and 17.76%, respectively. At the maximum power point (3600 r/min, full load), the output powers of T4W6-P10, T4W6-P20, and T4W6-P30 decreased by 5.01%, 10.03%, and 16.96%, respectively. The lower heating value of the mixed fuel decreased gradually with an increase of PODEn blending ratio^7,20,32^. According to the calculation, the lower heating values of T4W6-P10, T4W6-P20, and T4W6-P30 decreased by 6.81%, 13.27%, and 19.42%, respectively, compared with that of T4W6. The decrease extent of the lower heating value increased with an increase in the PODEn blending ratio, leading to larger decreases in the power and torque. Therefore, the blending ratio of PODEn should not be too high in actual use in order to avoid a lack of vehicle acceleration caused by the significant decline of the output power.

#### Fuel economy

**Figure 6:**
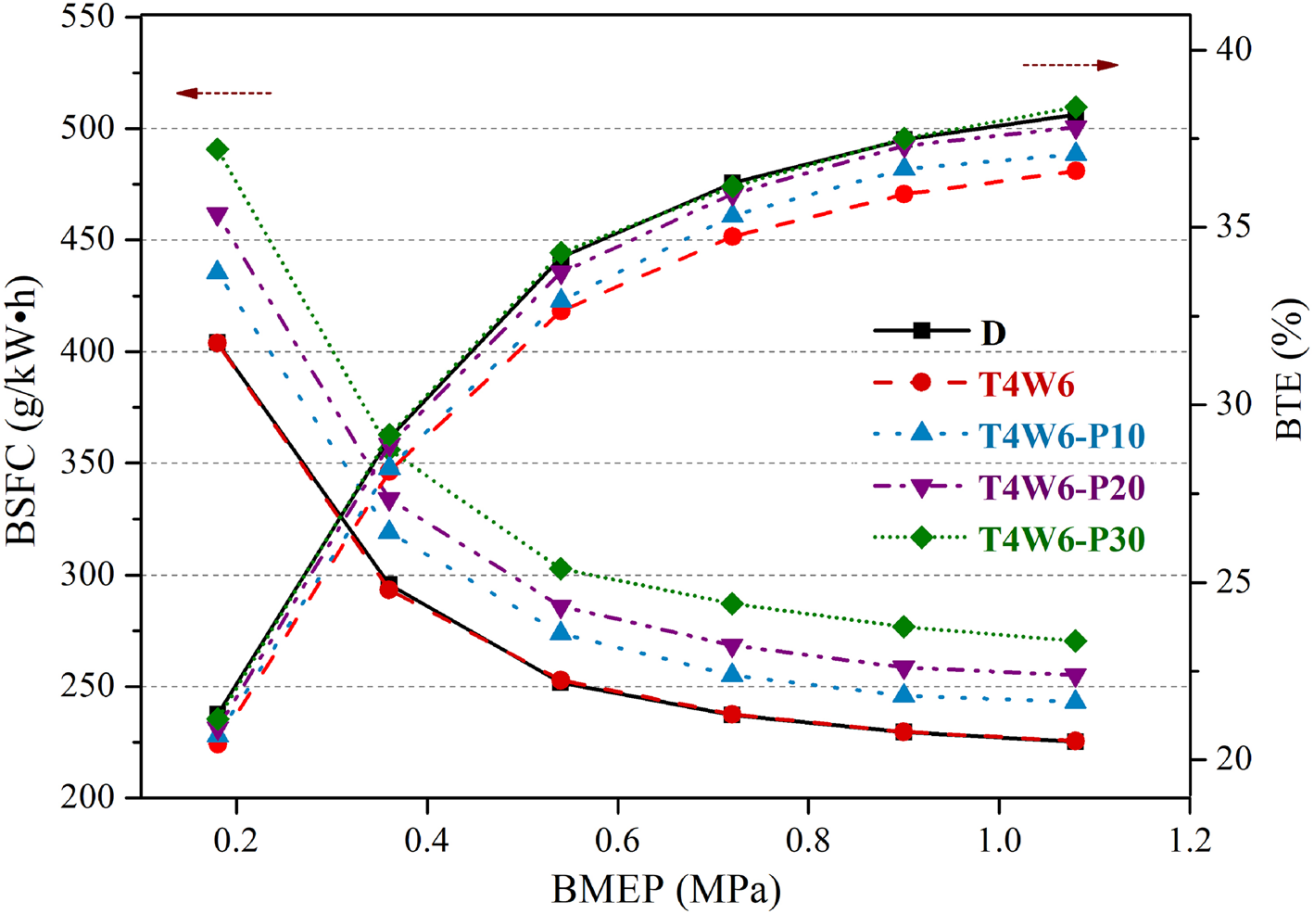
Effects of the PODEn blending ratio on the fuel economy of the diesel engine.

Figure 6 shows the effects of the PODEn blending ratio on the BSFC and BTE values of the diesel engine. It can be observed that at the same speed and load conditions, the BSFC increased gradually with an increase in the PODEn blending ratio. Due to the low lower heating value of PODEn, the lower heating values of T4W6-P10, T4W6-P20, and T4W6-P30 were 93.19%, 86.73%, and 80.58% of that of T4W6, respectively. Thus, the combustion heat of the blended fuels decreased. The corresponding torque outputs at the same BMEP conditions were the same, so the fuel injection quantities of the blended fuels increased, resulting in increases in the BSFC.

It can also be seen from Fig. 6 that blending with PODEn improved the BTE of the diesel engine, and the BTE increased gradually with an increase in the PODEn blending ratio. The BTEs of T4W6-P20 and T4W6-P30 were similar to that of petroleum diesel, and the BTE of T4W6-P30 was slightly higher than that of petroleum diesel in some conditions. When BMEP = 0.9 MPa, the BTE of T4W6-P10, T4W6-P20, and T4W6-P30 increased by 0.71%, 1.36%, and 1.57% compared to that of T4W6, respectively. It should be noted that the ignition delay was highly dependent on the fuel cetane number^21,22,32^. As presented in Table 4, adding PODE into T4W6 fuel increased the cetane number and thereby shortened the ignition delay. It also could be seen in previous studies that increasing PODEn blending ratios in blends, the combustion duration was also shortened, leading to a more concentrated heat release^20,21^. With an increase in the PODEn blending ratio, the oxygen content in the blended fuel increased, which reduced the fuel-rich regions and improved the combustion reaction speed^17,23,32^. All of these factors were conducive to improving the BTE of the diesel engine.

#### Emission characteristics

**Figure 7:**
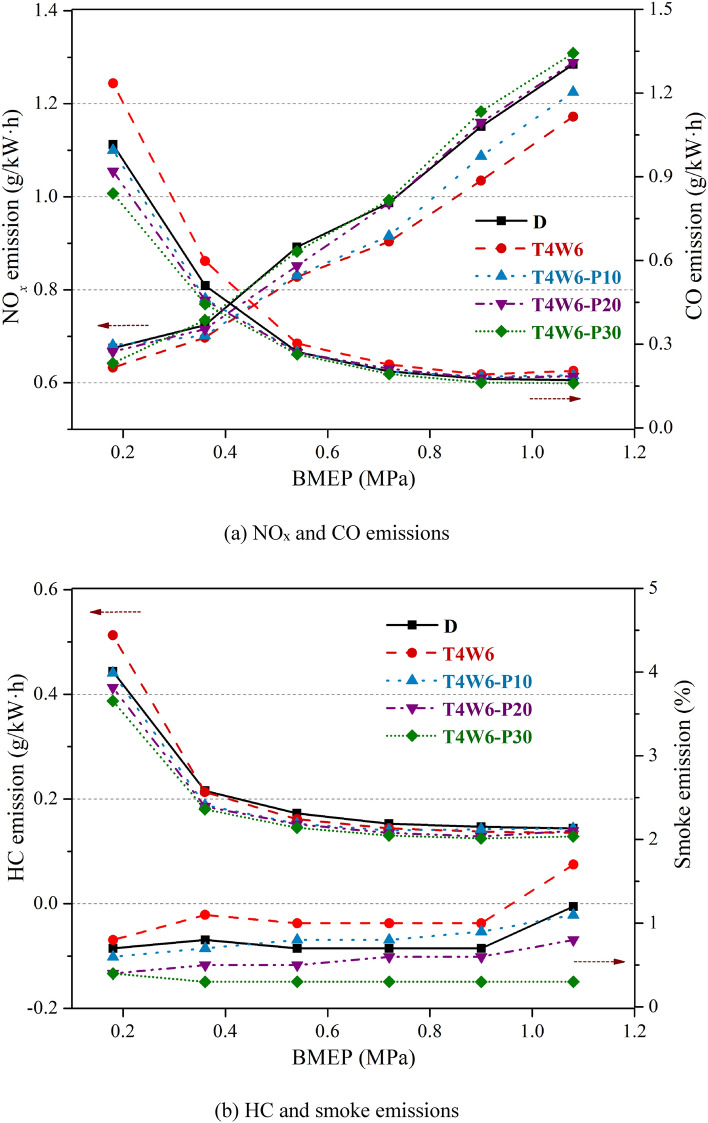
Effects of the PODEn blending ratio on the emission characteristics of the diesel engine.

Figure 7 presents the effects of the PODEn blending ratio on the emission characteristics of the diesel engine. It can be observed that the NOx emissions increased with an increase in the PODEn blending ratio. When BMEP = 0.9 MPa, the NOx emissions of T4W6-P10, T4W6-P20, and T4W6-P30 increased by 5.08%, 12.04%, and 14.34%, respectively. Higher oxygen content of PODEn increases the local oxygen concentration, which promotes the NOx formation. Meanwhile, the addition of PODEn slightly decreases the in-cylinder combustion temperature^20–22^, which inhibits the NOx generation. Under low engine loads, the in-cylinder combustion temperature is relatively low. Therefore, the oxygen-containing effect of PODEn on NOx emissions may predominate, leading to an increase in NOx emissions. At high-load conditions, the temperature in the cylinder was higher, and the NOx emissions increased rapidly with the increase in the oxygen concentration in the cylinder. Thus, the NOx emissions increased significantly at high-load conditions due to the high oxygen content of PODEn.

It can also be seen from Fig. 7a that blending with PODEn reduced the CO emissions to a certain extent, and the CO emissions decreased significantly with an increase in the PODEn blending ratio at medium and low loads. When BMEP = 0.18 MPa, the CO emissions of T4W6-P10, T4W6-P20, and T4W6-P30 decreased by 19.41%, 25.57%, and 31.67%, respectively. The ignition delay periods of the blended fuels shortened due to the high cetane number of PODEn. Therefore, the post-combustion ratio increased, which promoted the oxidation of CO. In addition, the high oxygen content of PODEn increased the air–fuel ratio and excess air coefficient, improving the combustion condition of the fuel-rich regions in the cylinder, which accelerated the oxidation rate of CO and then reduced the formation of CO emissions^17,20,32^.

It can be seen from Fig. 7b that blending PODEn with T4W6 could reduce the HC emissions. When BMEP = 0.18 MPa, the HC emissions of T4W6-P10, T4W6-P20, and T4W6-P30 decreased by 14.02%, 19.42%, and 24.42%, respectively. On the one hand, the cetane number of the blended fuel increased after blending with PODEn, the ignition performance of the fuel was improved, and the wall quenching layer around the injection fuel spray was reduced, which reduced the HC emissions. On the other hand, due to the low boiling point and high oxygen content of PODEn, the mixing and combustion speeds of fuel and gas were improved, which was conducive to the oxidation of HC emissions.

Figure 7b also demonstrates that blending with PODEn effectively improved the exhaust smoke of the diesel engine. When BMEP = 1.08 MPa, the smoke emissions of T4W6-P10, T4W6-P20, and T4W6-P30 were reduced by 35.29%, 52.94%, and 82.35% compared to those of T4W6, respectively. When the PODEn ratio was 30%, the smoke emissions were at a low level under all conditions. The combustion conditions in the fuel-rich regions during diffusion combustion were effectively improved due to the high oxygen content of PODEn. Moreover, PODEn had good evaporative and atomizing characteristics, which contributed to the formation of a combustible mixture and improved the combustion conditions of the fuel to a certain extent. Furthermore, there were no carbon–carbon (C–C) bonds in the PODEn molecules, which significantly reduced the quantities of olefins and polycyclic aromatic hydrocarbons (PAHs) generated during the combustion reaction, thereby further reducing the generation of smoke^18,19,28^. The above factors reduced the exhaust smoke emissions of the diesel engine significantly when PODEn were blended in the fuel.

## Conclusion

In this study, we examined the performance of a diesel engine fueled with FT diesel and hydrogenated diesel blends and evaluated the influence of blending with PODEn on the engine power performance, economy, and emission characteristics. The main conclusions are as follows:

1. The test diesel engine could be directly fueled with coal-based diesel fuels without any modification. Both the torque and power of the coal-based diesel blends were slightly lower than those of the petroleum diesel at high speeds, but the maximum reduction was only 2.71%. The BSFC values of the coal-based diesel blends were almost identical with that of the petroleum diesel. The BTE values of the coal-based diesel blends were slightly lower than that of the petroleum diesel, with the maximum reduction of 1.59%.
2. The emission characteristics of T5W5 were the most similar to that of petroleum diesel. T4W6 had low NOx emissions, with a maximum reduction of 11.18%. T6W4 had the highest CO and HC emissions, with maximum increases of 36.79% and 29.05%, respectively. The smoke emissions of T5W5 were slightly lower than those of petroleum diesel under certain conditions, while the smoke emissions of T4W6 and T6W4 were both higher than those of petroleum diesel.
3. Both the output torque and power decreased significantly when PODEn were blended into the fuel, and the decrease extent increased with the increase in the PODEn blending ratio. The BSFC and BTE gradually increased with an increase in the PODEn blending ratio, and the maximum increase in the BTE was 1.57%. Meanwhile, the emissions of NOx increased, but the emissions of CO, HC, and smoke decreased significantly with the increase in the PODEn blending ratio.

From the experimental results, it can be concluded that the engine fueled with coal-based diesel fuel showed a slightly decreased power and lower BTE while emitted higher NOx and smoke emissions. Adding PODEn into T4W6 improved the BTE and decreased smoke emissions at the expense of NOx emissions. In this work, the fuel injection strategies were followed by the original electric control unit, which was calibrated by the diesel fuel. Therefore, further work is required to evaluate the effects of fuel injection strategies like injection timing and multiple injection strategies on performance of coal-based diesel engines.

## Data Availability

The datasets used and analysed during the current study are available from the corresponding author on reasonable request.
